# Application of dynamic topic models to toxicogenomics data

**DOI:** 10.1186/s12859-016-1225-0

**Published:** 2016-10-06

**Authors:** Mikyung Lee, Zhichao Liu, Ruili Huang, Weida Tong

**Affiliations:** 1 NIH/National Center for Advancing Translational Sciences, Rockville, MD USA; 2 Division of Bioinformatics and Biostatistics, National Center for Toxicological Research, Jefferson, AR USA

**Keywords:** Dynamic topic model (DTM), Times-series gene expression, Toxicogenomics, TG-GATEs, Clustering, Topic modeling, Latent Dirichlet model

## Abstract

**Background:**

All biological processes are inherently dynamic. Biological systems evolve transiently or sustainably according to sequential time points after perturbation by environment insults, drugs and chemicals. Investigating the temporal behavior of molecular events has been an important subject to understand the underlying mechanisms governing the biological system in response to, such as, drug treatment. The intrinsic complexity of time series data requires appropriate computational algorithms for data interpretation. In this study, we propose, for the first time, the application of dynamic topic models (DTM) for analyzing time-series gene expression data.

**Results:**

A large time-series toxicogenomics dataset was studied. It contains over 3144 microarrays of gene expression data corresponding to rat livers treated with 131 compounds (most are drugs) at two doses (control and high dose) in a repeated schedule containing four separate time points (4-, 8-, 15- and 29-day). We analyzed, with DTM, the topics (consisting of a set of genes) and their biological interpretations over these four time points. We identified hidden patterns embedded in this time-series gene expression profiles. From the topic distribution for compound-time condition, a number of drugs were successfully clustered by their shared mode-of-action such as PPARɑ agonists and COX inhibitors. The biological meaning underlying each topic was interpreted using diverse sources of information such as functional analysis of the pathways and therapeutic uses of the drugs. Additionally, we found that sample clusters produced by DTM are much more coherent in terms of functional categories when compared to traditional clustering algorithms.

**Conclusions:**

We demonstrated that DTM, a text mining technique, can be a powerful computational approach for clustering time-series gene expression profiles with the probabilistic representation of their dynamic features along sequential time frames. The method offers an alternative way for uncovering hidden patterns embedded in time series gene expression profiles to gain enhanced understanding of dynamic behavior of gene regulation in the biological system.

**Electronic supplementary material:**

The online version of this article (doi:10.1186/s12859-016-1225-0) contains supplementary material, which is available to authorized users.

## Introduction

All biological processes including perturbation-responses are inherently dynamic. Investigating the temporal behavior of these dynamic processes is an important part of biological research. With the advancement of technology and reduction in cost, study of time-series gene expression has become routine [1]. The objectives of these types of research cannot be achieved without appropriate computational algorithms and methods. For example, a targeted perturbation like drug treatment activates or inhibits certain molecules in the cellular system in a transient or sustained manner; however, if we ignore these intrinsic dynamics of molecular changes due to lack of analysis techniques, we may miss out critical biological findings.

To analyze time-series gene expression profiles, several approaches have been used which can be divided into two classes. One of the classes is conventional clustering algorithms such as hierarchical, k-means clustering and self-organizing maps, which do not consider any dependencies between temporally successive profiles. In other words, even if we permute the order of time points, the results of these algorithms would not change. Additionally, another drawback of these approaches is the mutual exclusiveness of genes with respect to their involvement in biological processes responding to exposure. The second class of approaches is the clustering algorithms primarily designed to analyze time-series expression. For example, Aach and Church introduced dynamic time warping algorithm for the alignment of expression profiles in different time series [2]. Schliep introduced Hidden Markov Model widely used in speech recognition to consider time dependencies along sequential timeline of time series gene expression data [3]. These algorithms have been constantly under improvement [4–6]. Ramoni modeled the dynamics of genes by autoregressive equation and genes with the highest posterior probability from the same autoregressive equation were clustered together [7]. Additionally, there are software packages to analyze time-series gene expression data such as CAGED [7], STEM [8] and so on. CAGED applies regression analysis to cluster genes on the basis of their trajectories over multiple time points, while STEM first defines a set of representative temporal profiles, then assigns genes to one of several predefined temporal trajectories. These methodologies are more focused on clustering of genes showing similar expression patterns over time without an explicit consideration of sample-gene-time relationship.

In this study, we propose dynamic topic model (DTM) as a novel approach to cluster time-series gene expression profiles. DTM was originally developed by Blei to analyze the time evolution of topics in large document collections in the field of text mining [9]. DTM is an extension of Latent Dirichlet Allocation (LDA). LDA and other similar text mining methodologies haven been successfully applied to gene expression data analysis, some from our group [10–12], based on the similarity in data structure between text and gene expression. For example, Manuele et al. applied two different topic modeling approaches, PLSA (Probabilistic Latent Semantic Analysis) and LDA, to cancer classification using gene expression profiles [13]. Patrick et al. used a modified topic modeling technique to cluster drugs and genes [14]. Bing et al. applied a correspondence LDA model to discover microRNA regulated modules by identifying the microRNA and mRNA co-occurring frequently within the same latent variable [15]. Yu et al. applied topic modeling to discover functional modules with a biologically meaningful interpretation in RNA-Seq toxicogenomics data [16].

However, to the best of our knowledge, DTM has not been explored as an applicable method for the analysis of time-series gene expression profiles. It extended the static LDA to take into consideration time evolution that existed in a real document collection. Static LDA assumes that documents from the same set of topics are exchangeable, the probability of which is invariant to permutation. However, that assumption completely ignores one significant variable, i.e., time, that is present in documents organized according to sequential time where the topics evolve over time. DTM assumes that the topics associated with time, *t,* evolve from the topics associated with the previous time, *t*-1. It treats documents as mixtures of topics in which words are represented by the probability distribution across all time points. This representation can be analogously applied to biological systems since a biological component (topic) is different between the initial unstable stage, which is shortly after drug treatment, and the steady stage into which cellular system enters after a certain time period. In other words, the acting genes tend to evolve over time, consistent with the dynamic behavior of cellular and molecular effects after drug exposure changes over time. The advantage of using DTM is that it is a soft clustering technique which does not assume mutual exclusivity and permits multiple topic assignment with a probabilistic way to the same sample and gene, reflecting true biological complexity.

Here, we applied DTM to a set of time-series gene expression profiles generated by the Japanese Toxicogenomics Project [17] to investigate the dynamic feature of gene expression following drug treatment. Our study was built on the assumption that gene expression profiles resemble a set of documents, where each gene expression profile (a document) consists of mixtures of biological processes (that can be thought of as topics), and each biological process in turn consists of a set of genes (that can be thought of as the words used to present a topic). Based on the estimated latent topics, we clustered samples based on topic distributions followed by clustering of genes according to their contributions to each topic. Finally, the results provided us with a wide range of biological insights into how the genes’ activities are changed over time upon drug-perturbation.

## Materials and methods

### Dataset

The Japanese Toxicogenomics Project generated large-scale gene expression profiles for the same compounds tested in rat livers and kidneys as well as using both rat and human primary hepatocytes [17]. The datasets are organized in TG-GATEs. In its first phase, 131 compounds were profiled, most of which are drugs. Each compound was tested on three different assay platforms (i.e., in vitro assay, in vivo repeated dose study and in vivo single dose experiment) with a design including multiple doses and time points. In this study, we only utilized in vivo repeated dose experiments in which three doses (low, medium and high) and four time points (4, 8, 15, 29 days) were tested for 131 compounds. Among all dose levels, we selected high-dose repeated treatment under all-time points from rat livers, which consists of 3144 arrays (=131 compounds × 3 replicates × 4 timepoints × 2 doses). Further information about TG-GATEs can be found in Uehara et al. [17]. The TG-GATEs dataset used in this study was downloaded from CAMDA 2013 (http://dokuwiki.bioinf.jku.at/doku.php/start).

### Gene expression data processing

The probe-level microarray data were quantile normalized followed with mapping of a probe set into its corresponding genes [18], then multiple probes were summarized into one corresponding gene’s intensity ratio using FARMS [19]. Next, we generated a “document” for each drug-time condition; it contained “words”, each represented genes differentially expressed by comparing the treated against the matched control. Thus, each document was then represented by its differentially expressed genes (DEGs) (words). A total of 514 documents (131 compounds × 4 time points) were generated; each document was tagged with each time point, such as 4, 8, 15 and 29 days. A total of 12,088 genes were present in this experiment. We considered the same gene with different transcriptional directions (i.e., up and down) as two different words, leading to a corpus of 24,176 words. The frequency of a word appearing in each document was determined by multiplying 100 to the fold change of DEGs.

### Model representation

All variants of LDA are probabilistic, which usually involves a series of processes to determine the optimal parameters to maximize the posterior probability of the observed data. In static LDA, α and β_k_ are the Dirichlet prior parameters on the topic distributions over document and the word distribution over topic *k*, respectively. Different from a static LDA, DTM adopts logistic normal distribution for two prior distributions (topic per document and word per topic) and hence is more complex compared to static LDA, which assures conjugacy between prior and posterior distributions. Specifically, static LDA assumes that the words of each document are independently drawn from a mixture of multinomial. However, this implicit assumption of independency is not appropriate, because the topic (a set of words) in a document collection evolves over time. Our goal is to explicitly address the dynamics of the underlying topics as a function of sequential time. DTM provides a solution to this problem by assuming that topics at time *i* evolved from the topics at time *i-1* with the reflection of real organization of document collections*.* DTM assumes that the data is divided by time slice, modeling the documents of each slice with a static topic model, where the topics associated with slice *t* evolve from the topics associated with slice *t* – 1. In a static LDA model, it assumes that the topic-specific word distributions are drawn from a Dirichlet distribution. However, DTM does not assume Dirichlet distribution to approximate posterior inference, the word distributions over multiple time points are chained by Gaussian distribution. Due to the nonconjugacy of the Gaussian and multinomial models, Blei applies variation approximations such as Kalman filters and nonparametric wavelet regression to approximate posterior inference.

In this study, the open-source DTM C^++^ package was applied from the author’s website (https://www.cs.princeton.edu/~blei/topicmodeling.html). The modeling results include two different distributions: multinomial distribution over topics for each document and multinomial distributions over words for each time point associated with each topic. In our analysis, the number of topics was heuristically determined by closely examining two hyperparameters*, alpha* and *top_chain_var* which defines the number of topics. Specifically, *alpha* controls the shape of the topic distribution of a sample. A smaller *alpha* results in each document to be more probabilistically associated with fewer topics. The *top_chain_var* determines how similar topics would be over multiple time points. A smaller *top_chain_var* leads to similar word distributions over multiple time points. In our study, we have tested several parameter settings for *alpha* and *top_chain_var* and found that the varied values do not have a significant effect on our interpretation of the sample clustering results and topic distribution over time points. Thus, choose the default value of (alpha = 0.01, top_chain_var = 0.005) and, at this condition, we feel that the choice of 20 topics is sufficient to balance between extreme generalization of the model and maximizing the chance of an informative discovery.

### Clustering samples and genes

After building a probabilistic model for our observed temporal DEGs using DTM, two distributions (matrix) were generated: topic distribution over document and a series of word distributions over multiple time points for each topic. The former includes the conditional probability of each topic given a sample, *P(T|D)*. This probability is a signature of the sample, which can be used to assess sample similarities. The latter represents the conditional probability of each gene given a topic at a particular time point, *P(W|T)*_*time*_, indicating which genes are important to a given topic in a particular time point. As we have four time points (4, 8, 15 and 29 days), four different *P(W|T)* were obtained, i.e., *P(W|T)*_*4days*_, *P(W|T)*_*8days*_, *P(W|T)*_*15days*_, *and P(W|T)*_*29days*_. First, to group documents, each document was assigned to the topic with the largest conditional probability value of *P(T|D)*. The other distribution, *P(W|T)*_*time*_ was used for clustering genes. Since DTM is designed to cluster words co-occurring frequently across whole documents, the genes with a high rank in the same topic are likely involved in the same biological process. To take advantage of this information, functional pathway analysis was performed for each topic using the Fisher’s exact test with data from Kyoto Encyclopedia of Genes and Genomes (KEGG) (http://www.genome.jp/kegg/), and Gene Ontology (GO) (http://geneontology.org/). Above all, the strongest benefit of DTM is its ability to monitor the behavior of the genes at the given time points and thus aid in investigating the significantly active genes at each time point. Each gene was ranked for each time point, and functional analysis was conducted for the top 300 genes according to their rank.

## Results and discussion

This study consists of several steps: (1) generation of documents of DEG lists for each compound at each time point; (2) Building a generative probabilistic model using DTM to maximize the posterior probability of observed temporal DEGs; (3) Assignment of the topic with largest conditional probability value to each compound-time condition; (4) ranking DEGs according to their conditional probability of each topic and assessment of topic evolution over time (4) topic analysis in the biological context. From these procedures, we obtained two outputs, one of them is *P(T|D),* the topic distributions for a given compound-time condition, and the other is *P(W|T)*_*time*_, a series of distributions over genes at multiple time points for each topic.

### Study of topics

DTM provides the distribution of topics for a given compound-time condition, *P(T|D)*. This can be used for the assessment of the association between a specific condition and a specific topic. We used this statistical probability to group the conditions by connecting them with topics. These results are provided in Additional file 1: Table S1 that includes Mode of Action (MoA) and therapeutic category information for the 131 drugs. We found that drugs with the same MoA category tend to be highly associated with the same topic. Specifically, all of the eight drug-time condition associated with topic 5 are PPAR α agonists (WY-14643 and fenofibrate) at all four time points, indicating that these drugs have consistent DEGs (vocabularies) across the whole time points and that the PPARα agonist action is not time sensitive compared to other compounds. Other than topic 5, topic 1 is also found to be associated with PPARα agonists (clofibrate and gemfibrozil) at all four time points. However, unlike topic 5, topic 1 also includes several other drugs that are not PPARα agonists, such as amiodarone, aspirin, bendazac, benzbromarone, chloramphenicol and simvastatin. Benbromarone is not a PPARα agonist; but it is known to have a high binding affinity for PPARα, showing potential as a PPARα agonist [20]. It has also been reported that the adverse effect of amiodarone is related to expression of PPARα target genes, implying the possibility of PPARα as one of its off-targets [21]. In addition to these two drugs, the relationship between PPARα and aspirin and simvastatin has also been studied [22, 23]. Another example is topic 17, which showed high selectivity, 68 % (21 / 31) for COX inhibitors, including non-steroidal anti-inflammatory drugs such as ibuprofen, mefenamic acid, phenylbutazone, sulindac, diclofenac, naproxen, nimesulide and indomethacin. We also found that some of the topics are associated with only a single drug. For instance, all the samples over four time points of ethambutol, thioacetamide and ethionine were assigned topic 4, 12 and 15, respectively, showing their distinct drug effect with less sensitivity to time.

### Associating topics with functional pathways

**Fig. 1:**
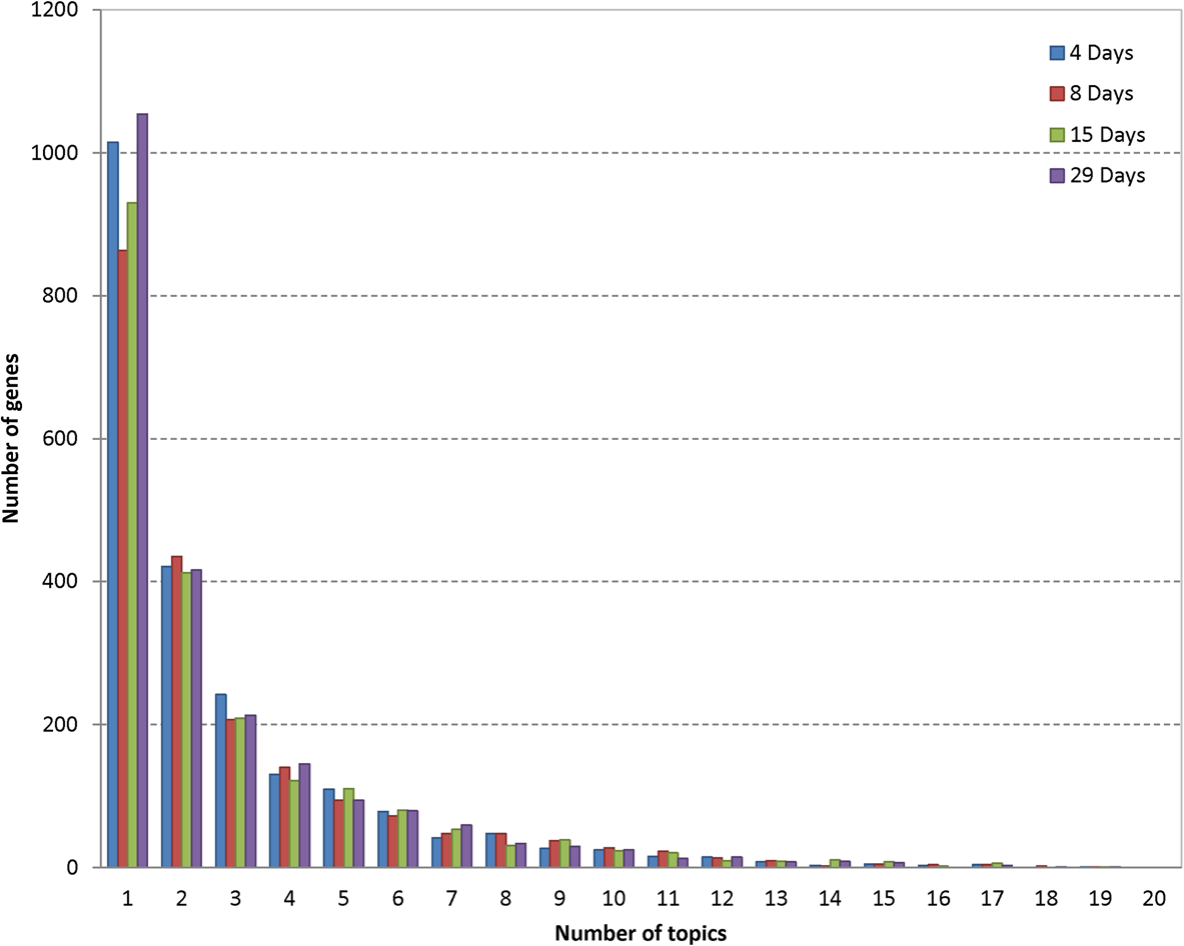
Distribution of genes across all the topics

**Fig. 2:**
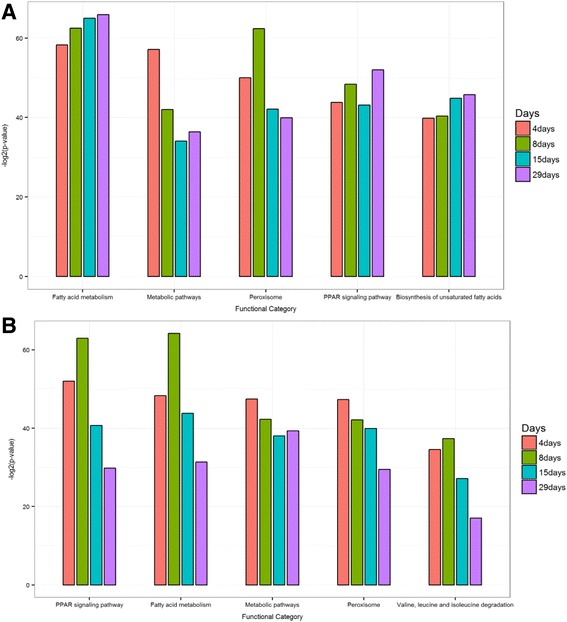
Functional pathway analysis result for Topic 1 and Topic 5. **a** Topic 1’s top five functional pathways significant over 4 time points. **b** Topic 5’s top five functional pathways significant over 4 time points

A second product of DTM is the distribution of words (DEGs) for a particular topic, *P(W|T)*. Especially, in our analysis, four different *P(W|T)*_*4days*_, *P(W|T)*_*8days*_, *P(W|T)*_*15days*_, *P(W|T)*_*29days*_ were derived. This probability can be interpreted as the contribution of a gene to a particular topic at a certain time point. The most associated 10 genes at 4 days for each topic is provided in Table 1 as an example. First, representative genes for each topic were extracted by selecting genes ranked within 300 at four time points (Additional file 2: Table S2). One of the strength of DTM is that it does not assume mutual exclusivity, leading to a systematic interpretation of genes involved in multiple pathways. Figure 1a shows the frequency of each gene across 20 topics for four time points. Over the whole set of time points, half of the genes only appear once while the remaining genes are present in multiple topics. We found that the down regulation of *stac3* was associated with the largest number (19) of topics across all of the four time points. *Stac3* (SH3 And Cysteine-Rich Domain-Containing Protein) is well known to be highly expressed and control the cell cycle in skeletal muscle while little is known about its involvement in liver function. To determine which biological processes were over-represented in a particular topic, we searched KEGG and GO with the top 300 ranked genes at each time point for each topic and used the Fisher’s exact test to assess the significance of association (Additional file 3: Table S3). As expected, most of the top biological processes were metabolism of xenobiotics by cytochrome P450 which is a major enzyme family catalyzing the oxidative biotransformation of most drugs [24]. Figure 2 shows topic 1 and topic 5’s top pathways significance distribution across four time points as examples. All of the top 300 ranked genes in topic 1 and 5 had PPAR signaling pathway as the top functional category except in the 4-day time-point of topic 1. Fatty acid metabolism was found to be the top ranked category at 4 days in topic 1. Nevertheless fatty acid metabolism is also well known to be associated with PPAR signaling [25]. Additionally, the top biological process of topic 4 is ribosome, of which members are composed of EBU_Rat_4day, EBU_Rat_8day, EBU_Rat_15day, EBU_Rat_29day and MP_Rat_4day. Ethambutol (EBU) is a medication used to treat tuberculosis, which is known to possibly target ribosome [26].

**Table 1:** Each topic is composed a set of genes. Genes are ranked according to the probability of topic-gene matrix. The table shows the top 10 genes at the time point of 4 days

Topic1	Topic2	Topic3	Topic4	Topic5
Acot1_up	Lcn2_up	Dhrs7_down	Trib3_up	Acot1_up
Vnn1_up	S100a8_up	Akr1b7_up	Fgf21_up	Fabp3_up
Aig1_up	S100a9_up	Slc22a8_down	Ddit3_up	Cpt1b_up
Ehhadh_up	LOC360228_up	Rbp7_up	Pycr1_up	Hdc_up
Eci1_up	Spink3_up	A1bg_up	Nupr1_up	Vnn1_up
Ech1_up	RGD1307603_down	Car3_down	Acot1_up	Aig1_up
Cyp4a1_up	Lbp_up	Ust5r_down	Phgdh_up	Acot3_up
Acaa1a_up	A2m_up	Rdh2_down	Gsta5_up	Aqp7_up
Acot2_up	Stac3_down	Gsta5_up	Asns_up	RGD1305928_up
Aldh1a1_up	Cxcl1_up	Cyp3a9_up	Akr7a3_up	Stac3_down
Topic6	Topic7	Topic8	Topic9	Topic10
Cyp2c11_down	Stac3_down	Car3_down	Gstm3_up	Gsta5_up
Car3_down	Aldh1a1_up	Dhrs7_down	Stac3_down	Ces2c_up
Cyp2a2_down	Ces2c_up	Cyp2c11_down	Lcn2_up	Aldh1a1_up
Ust5r_down	Gsta5_up	Stac3_down	Car3_down	Aldh1a7_up
Hao2_down	Akr7a3_up	Ust5r_down	Zfp354a_down	Akr7a3_up
Cyp3a2_down	Scd1_down	Kynu_down	Sds_down	Stac3_down
Cyp2d3_down	Mgmt_up	Sult1c3_down	Lbp_up	Dhrs7_down
Slc10a1_down	Oat_down	Sult1e1_down	Gpnmb_up	Abcc3_up
Sult1e1_down	Cdkn1a_up	Aldh1a7_up	Epcam_up	Ugt2b1_up
Lipc_down	Ccng1_up	Slc22a8_down	LOC360228_up	Cyp1a1_up
Topic11	Topic12	Topic13	Topic14	Topic15
Gstp1_up	Stac3_down	Oat_down	Stac3_down	RGD1584021_up
Stac3_down	Oat_down	Gstm3_up	Cyp1a1_up	Isg15_up
Akr7a3_up	Dhrs7_down	Stac3_down	Scd1_down	Gstm3_up
Oat_down	Lcn2_up	Pln_up	Kif20a_down	Fads1_down
Ccrn4l_up	Gstm3_up	Aldh1a1_up	Ccnb1_down	Ca2_up
Pycr1_up	Cyp1a2_down	Rbp7_up	Ect2_down	Fads2_down
Inmt_down	Fam25a_up	Ces2c_up	Ube2c_down	Slc6a13_down
Tmed3_up	Sds_down	Ccng1_up	Nusap1_down	Rsad2_up
Fkbp11_up	Anxa2_up	Pbk_up	Cdk1_down	LOC100361444_up
Cdk1_up	Aldh1a1_up	Inmt_down	Prc1_down	Ftcd_down
Topic16	Topic17	Topic18	Topic19	Topic20
Car3_down	A2m_up	Akr1b7_up	Scd1_down	Pglyrp1_up
Gstm3_up	Lcn2_up	Isyna1_up	Zfp354a_up	Cyp1a2_down
Stac3_down	Stac3_down	Stac3_down	Gsta5_up	Npy_up
Aldh1a1_up	Serpina7_up	Aldh1a1_up	Dhrs7_down	Pln_up
Trib3_up	Cxcl1_up	Spink3_down	Ube2c_down	Ces2c_up
Aldh1a7_up	Lbp_up	Scd1_down	RGD1309362_down	Rbp7_up
Gsta5_up	LOC360228_up	A2m_down	Aldh1a7_up	Lcn2_up
Cyp4b1_up	Dhrs7_down	Cyp17a1_up	Aldh1a1_up	Tspan8_up
Spink1_down	Fgl1_up	Aldh1a7_up	Hamp_up	LOC299282_down
Hal_down	S100a9_up	Cyp2c11_down	Gstt3_up	S100a10_up

### Assessment of word evolution over time

**Fig. 3:**
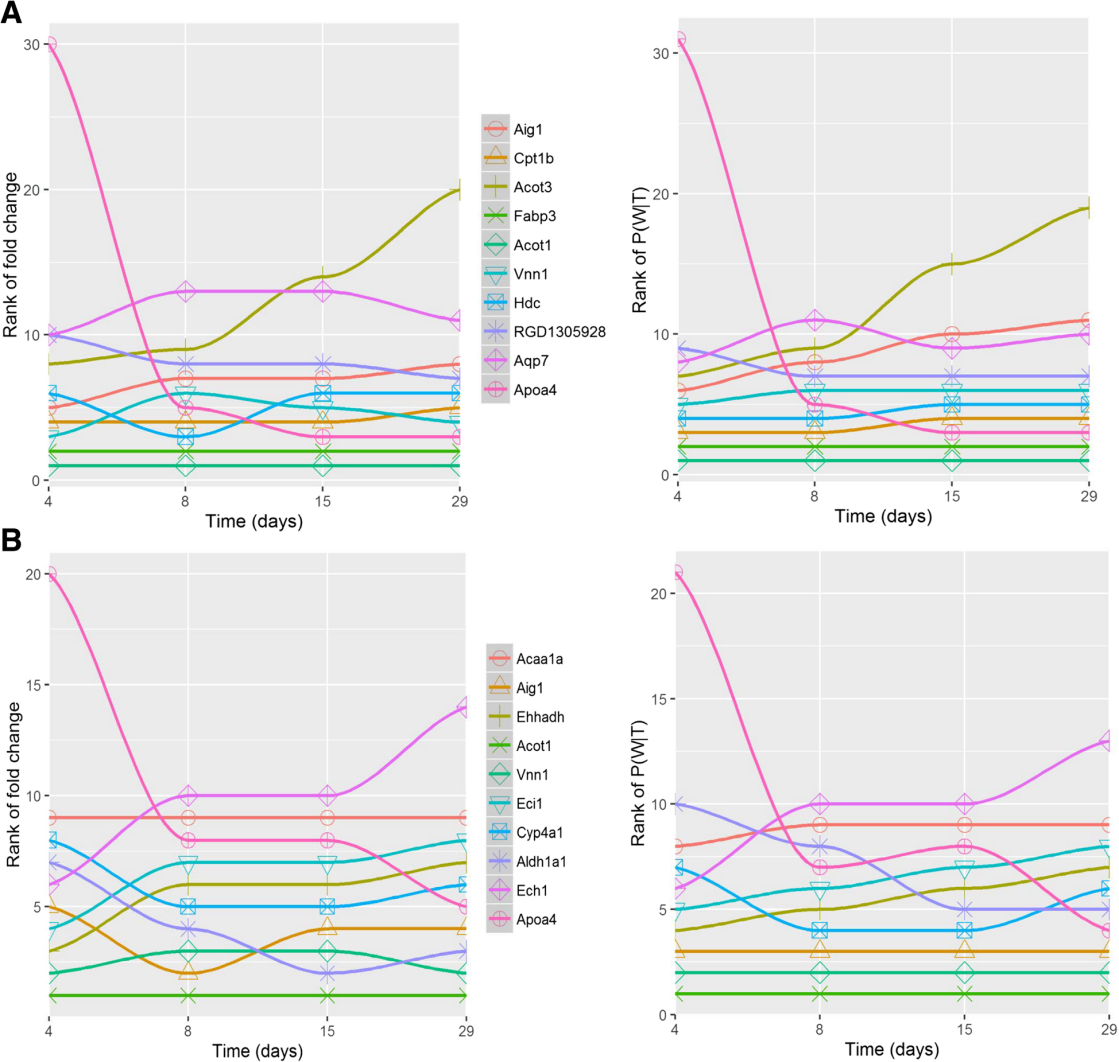
Comparison between the ranks from DTM and the original gene expression fold changes. Left panel plots the rank of original absolute fold change of the top 10 ranked genes while right panel plots the rank of *P(W|T)* estimated from DTM. **a** and **b** show topic 5 and topic 1, respectively

In static topic modeling, we only derive a single *P(W|T)* regardless of time; however, DTM yields multiple conditional distributions, *P(W|T)*_*4days*_, *P(W|T)*_*8days*_, *P(W|T)*_*15days*_, *P(W|T)*_*29days*_ for each time point that can offer more information for biological interpretation. From these probabilities, the dynamic nature of genes was investigated. The results from DTM and the original fold changes are compared in Fig. 3, where the left panel plots the rank of original absolute fold change of the highly ranked genes in topics 5 and 1 (panels A and B), while the right panel shows the rank of *P(W|T)* estimated from the DTM across four time points. As expected, in Topic 5, *Acot1* is most highly up-regulated over the whole set of time points followed by *Fabp3. Acot1* (Acyl-CoA thioesterase I) is an enzyme that hydrolyzes long-chain acyl-CoAs to the free fatty acid and coenzyme A and is widely known to be the target of PPARα agonists [27]. *Fabp3* is a well-known biomarker for skeletal muscle toxicity while little is known about its function in liver [28]. Even though four PPARα agonists were associated with topic 5, only two of them (WY-14643 and fenofibrate) triggered enormous mRNA increase in *Fabp3*. Also, *Fabp3* has been reported to cause drug induced liver injury. The drastic change of *Apoa4* was observed from 8-day rather than the initial stage right after drug treatment, which was reflected by *P(W|T). Apoa4* is known to be one of the apolipoproteins that bind lipids to form lipoproteins and transport the lipids out of the liver.

### Comparison with other bi-clustering methods

**Fig. 4:**
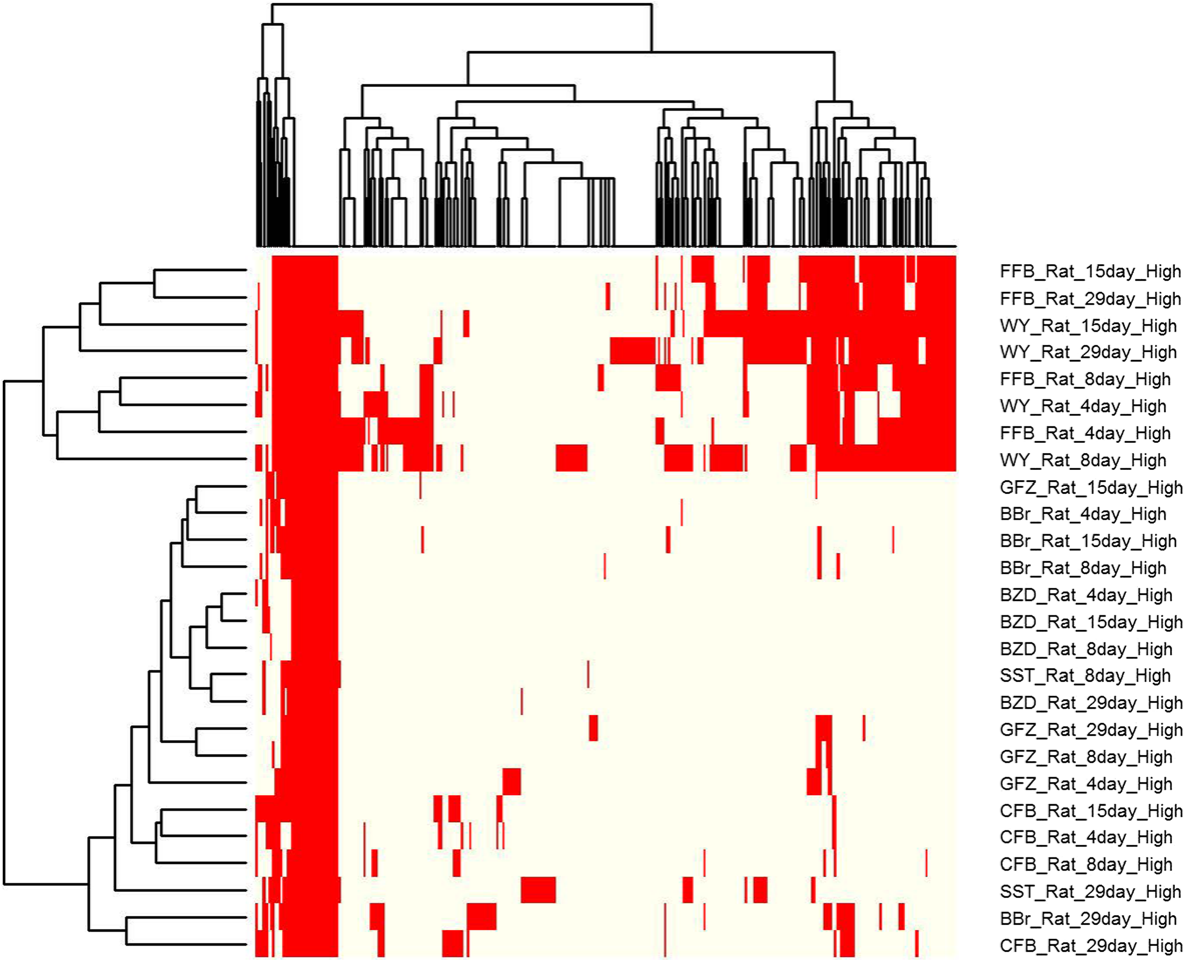
Clustering of drug-time condition by GO categories. A total of 338 distinct GO categories are enriched for 26 drug-time conditions which are identified from ISA. If a condition is enriched with a certain GO category is colored with red, otherwise it is colored with ivory

To demonstrate the advantage of DTM, we tested conventional bi-clustering algorithms with the same data matrix used for DTM. The iterative signature algorithm (ISA) is one of the most widely used bi-clustering algorithms [29]. We utilized the R package named *eisa* to generate co-expression modules. The thresholds of standard deviations from the mean gene expression were varied from 2.5 to 5 by 0.1 along both rows and columns, keeping only genes and samples that showed expressions levels that exceeded the given threshold. Among them, totals of 25, 7, 8, 3, 3 and 2 modules were identified at the thresholds of 2.5, 3, 3.5, 4, 4.5 and 5, respectively. At all thresholds, clusters of PPARα agonists were identified. When a threshold of 2.5 was applied, the ISA yielded one co-expression module representing PPARα agonists, which included 528 genes and 26 drug-time conditions including seven different drugs namely WY-14643, benzbromarone, benziodarone, clofibrate, fenofibrate, gemfibrozil and simvastatin. Even though four drugs among seven (WY-14643, clofibrate, fenofibrate and gemfibrozil) were PPARα agonists, they showed discernible enriched GO patterns as illustrated in Fig. 4. To be more specific, we analyzed the DEGs of the 26 drug-time conditions for enrichment in GO processes, and clustered them based on the similarity in their activity patterns in terms of their enriched GO processes (Fig. 4). We found that these conditions formed two distinct groups. One of the groups was found to be composed of WY-14643 and fenofibrate with the rest of the drugs separated from them. This difference was found in our DTM results where these conditions were assigned to two topics, topic 1 and 5. In contrast, these 26 conditions formed a single group when using ISA with 2.5 as the threshold. When we select a higher threshold, specifically, at the threshold of 3.7, there were two PPARα agonists. One of them is composed of BBr_Rat_4day, BBr_Rat_4day, BBr_Rat_4day, BBr_Rat_4day, CFB_Rat_8day and FFB_Rat_8day while the other is consisting of all samples of WY-14643 and fenofibrate. At any threshold, we could not find bi-clusters to separate them into two groups in an accurate manner as illustrated in Fig. 4. At the threshold of 3.8, a module containing two drugs (WY-14643 and fenofibrate) was identified while the rest of the PPARα agonists were not detected. This implies that DTM can assess the qualitative change without the over-representation of quantitative change to encompass even the modest change in the data through model based approach. The other clustering algorithm we tested is a dynamic tree cut, which uses a dendrogram to identify clusters and does not need cluster size defined in advance [30]. We adjusted the parameter maxCoreScatter for it to return a cluster size similar to our DTM result. We set the method and deepsplit as hybrid and 4, respectively. When we set the maxCoreScatter as 0.615, the cluster size was 21, which is comparable to our DTM result. Like the ISA algorithm, only one composite group was identified, instead of two distinct groups of PPARα agonists being identified separately. Even when we increased the cluster size up to 38 by adjusting the maxCoreScatter, the two different groups were not accurately identified. These results are presented in Additional file 4: Table S4. In addition to its strength in sample clustering, DTM not only showed good performance in clustering genes but also provided valuable information on their dynamics. For example, *Fabp3* is highly expressed upon exposure to WY-14643 and fenofibrate, which is identified in topic 5 (Fig. 3a) but not as significantly expressed in the presence of other PPARα agonists. When ISA is applied, *Fabp3* is included in every PPARα agonist relevant cluster. More importantly, while the general bi-clustering algorithm does not consider the time sequence, DTM models the activity changes of genes according to time relapse as presented in Fig. 3.

## Conclusions

To supplement the drawbacks of traditional clustering algorithms, DTM was explored as one of the model based algorithms for the analysis of time-series gene expression data, which has not been applied previously to our best knowledge. Therefore, our study is significant as a pilot study that explores the feasibility of applying a text mining approach to time-series biological datasets. As a result of our investigation, we identified hidden patterns embedded in time series toxicogenomics. From the topic distribution for each document, a number of drugs were successfully clustered by their shared MoA, for example, PPARɑ agonists and COX inhibitors. The biological meaning underlying each topic was interpreted using diverse sources of information such as functional analysis of the pathways and therapeutic uses of the drugs, which could provide a better understanding of drug perturbation mechanisms. Additionally, we found that sample clusters produced by DTM are much more coherent in terms of functional categories than the ones from traditional clustering algorithms. Above all, time specific activity distribution according to each sequential time provided tremendous opportunity to uncover the underlying toxicological dynamic changes. In summary, our study found that DTM has several distinct advantages. Firstly, it can reduce data dimension very effectively in terms of the latent variable (i.e., topic), with the assumption of time-dependency present in toxicogenomics. Secondly, it also allows samples and genes to be associated with multiple topics in an intuitive probabilistic manner without the mutual exclusivity assumption, reflecting the complexity of real biological system. Most importantly, topic dynamics over time relapse could provide new biological insights into the evolution of gene regulation.

## Additional files

Additional file 1: Table S1. Information for each topic, including Mode of Action (MoA), therapeutic category, and DILI annotation. (XLSX 29 kb)
Additional file 2: Table S2. Top 300 genes that represent each topic at 4 time points. (XLSX 166 kb)
Additional file 3: Table S3. Functional analysis results for each topic at 4 time points: Fisher’s exact test with a *p* value cut-off of 0.05 was used in KEGG and GO. (XLSX 82 kb)
Additional file 4: Table S4. Sample clustering results using dynamic tree cut. (XLSX 42 kb)

## References

[1] Bar-Joseph Z, Gitter A, Simon I (2012). Studying and modelling dynamic biological processes using time-series gene expression data. Nat Rev Genet.

[2] Aach J, Church GM (2001). Aligning gene expression time series with time warping algorithms. Bioinformatics.

[3] Schliep A, Schönhuth A, Steinhoff C (2003). Using hidden Markov models to analyze gene expression time course data. Bioinformatics.

[4] Smith AA, Vollrath A, Bradfield CA, Craven M (2008). Similarity queries for temporal toxicogenomic expression profiles. PLoS Comput Biol.

[5] Yoneya T, Mamitsuka H (2007). A hidden Markov model-based approach for identifying timing differences in gene expression under different experimental factors. Bioinformatics.

[6] Zhao C, Hua J, Bittner ML, Ivanov I, Dougherty ER (2012). Identifying mechanistic similarities in drug responses. Bioinformatics.

[7] Ramoni MF, Sebastiani P, Kohane IS (2002). Cluster analysis of gene expression dynamics. Proc Natl Acad Sci.

[8] Ernst J, Bar-Joseph Z (2006). STEM: a tool for the analysis of short time series gene expression data. BMC Bioinformatics.

[9] Blei DM, Lafferty JD. Dynamic topic models. In: Proceedings of the 23rd international conference on Machine learning; 1143859. Pittsburgh: ACM; 2006. p. 113–20.

[10] Lee M, Huang RL, Tong WD (2016). Discovery of transcriptional targets regulated by nuclear receptors using a probabilistic graphical model. Toxicol Sci.

[11] Chung MH, Wang YP, Tang HL, Zou W, Basinger J, Xu XW, Tong WD. Asymmetric author-topic model for knowledge discovering of big data in toxicogenomics. Front Pharmacol. 2015;6:81. 10.3389/fphar.2015.00081 PMC4403303 25941488

[12] Lee M, Liu ZC, Kelly R, Tong WD. Of text and gene - using text mining methods to uncover hidden knowledge in toxicogenomics. BMC Syst Biol. 2014;8:93. 10.1186/s12918-014-0093-3 PMC4236689 25115450

[13] Bicego M, Lovato P, Oliboni B, Perina A. Expression microarray classification using topic models. In: Proceedings of the 2010 ACM Symposium on Applied Computing; 1774415. Sierre: ACM; 2010. p. 1516–20.

[14] Flaherty P, Giaever G, Kumm J, Jordan MI, Arkin AP (2005). A latent variable model for chemogenomic profiling. Bioinformatics.

[15] Liu B, Liu L, Tsykin A, Goodall GJ, Green JE, Zhu M, Kim CH, Li J (2010). Identifying functional miRNA–mRNA regulatory modules with correspondence latent dirichlet allocation. Bioinformatics.

[16] Yu K, Gong B, Lee M, Liu Z, Xu J, Perkins R, Tong W (2014). Discovering functional modules by topic modeling RNA-seq based toxicogenomic data. Chem Res Toxicol.

[17] Uehara T, Ono A, Maruyama T, Kato I, Yamada H, Ohno Y, Urushidani T (2010). The Japanese toxicogenomics project: application of toxicogenomics. Mol Nutr Food Res.

[18] Dai M, Wang P, Boyd AD, Kostov G, Athey B, Jones EG, Bunney WE, Myers RM, Speed TP, Akil H (2005). Evolving gene/transcript definitions significantly alter the interpretation of GeneChip data. Nucleic Acids Res.

[19] Hochreiter S, Clevert D-A, Obermayer K (2006). A new summarization method for affymetrix probe level data. Bioinformatics.

[20] Kunishima C, Inoue I, Oikawa T, Nakajima H, Komoda T, Katayama S. Activating effect of benzbromarone, a uricosuric drug, on peroxisome proliferator-activated receptors. PPAR Res. 2007;2007:36092. 10.1155/2007/36092 PMC2233808 18274627

[21] McCarthy TC, Pollak PT, Hanniman EA, Sinal CJ (2004). Disruption of hepatic lipid homeostasis in mice after amiodarone treatment is associated with peroxisome proliferator-activated receptor-α target gene activation. J Pharmacol Exp Ther.

[22] Seo M, Inoue I, Ikeda M, Nakano T, Takahashi S, Katayama S, Komoda T. Statins activate human PPARalpha promoter and increase PPARalpha mRNA expression and activation in HepG2 cells. PPAR Res. 2008;2008:316306. 10.1155/2008/316306 PMC2610383 19125197

[23] Yiqin Y, Meilin X, Jie X, Keping Z (2009). Aspirin inhibits MMP-2 and MMP-9 expression and activity through PPARα/γ and TIMP-1-mediated mechanisms in cultured mouse celiac macrophages. Inflammation.

[24] Zanger UM, Schwab M (2013). Cytochrome P450 enzymes in drug metabolism: regulation of gene expression, enzyme activities, and impact of genetic variation. Pharmacol Ther.

[25] Poulsen LC, Siersbæk M, Mandrup S (2012). PPARs: fatty acid sensors controlling metabolism. Semin Cell Dev Biol.

[26] Lin Y, Li Y, Zhu Y, Zhang J, Li Y, Liu X, Jiang W, Yu S, You X-F, Xiao C (2012). Identification of antituberculosis agents that target ribosomal protein interactions using a yeast two-hybrid system. Proc Natl Acad Sci.

[27] Dongol B, Shah Y, Kim I, Gonzalez FJ, Hunt MC (2007). The acyl-CoA thioesterase I is regulated by PPARα and HNF4α via a distal response element in the promoter. J Lipid Res.

[28] Pritt ML, Hall DG, Recknor J, Credille KM, Brown DD, Yumibe NP, Schultze AE, Watson DE (2008). Fabp3 as a biomarker of skeletal muscle toxicity in the rat: comparison with conventional biomarkers. Toxicol Sci.

[29] Bergmann S, Ihmels J, Barkai N (2003). Iterative signature algorithm for the analysis of large-scale gene expression data. Phys Rev E Stat Nonlin Soft Matter Phys.

[30] Langfelder P, Zhang B, Horvath S (2008). Defining clusters from a hierarchical cluster tree: the Dynamic Tree Cut package for R. Bioinformatics.

